# Advertisers, &c

**Published:** 1889-01

**Authors:** 


OXYGEN APPARATUS.—We ask especial attention to the advertisement of the Oxygen apparatus, sold by THE AMERICAN OXYGEN ASSO 119 East 28th St., New York. This apparatus will prove an attractive feature in the office of the progressive practitioner. An illustrated catalogue and full explanation of the method of using the apparatus etc., may be obtained from the above House. See the advertisement.

PARKE, DAVIS & CO —This enterprising and accommodating House has recently forwarded us for inspection and trial the following preparations, to wit: *Fluid Extract Cascara Sagrada*, *Syrup of Yerba*, *Aromatic Santa; Cascara Cordial*, prepared exclusively for physicians, presenting the cascara in a most neat and palatable form; the Concentrated Tinct. of *Lippea Mexicana Leaves*, a new and important demulcent and expectorant; the *Syrup Trifolium Compound*, a superior alterative especially in syphilitic affections.

WE call the attention of our readers to the advertisement of Messrs R. A. Robinson & Co., Louisville Ky., which will be found on another page of this issue. This firm was established forty-five years ago, and enjoys a wide-spread reputation as a sound, honest, reliable business house. Their preparations are believed to be all they claim for them.

NEW APPLIANCE.—Please examine the advertisement of J. Elwood Lee & Co., in reference to their patented metalic Splints. It would certainly be a great convenience and satisfaction to the practitioner to have ever at hand a set of these splints, so that he could easily and without delay dress a fracture. We have received samples of these splints and find them excellent.

WM. R. WARNER & Co.—The polite and gentlemanly Agent of this staunch and reliable House of Philadelphia, recently called at our Sanctum with beautiful samples of their excellent preparations. Among them their Bromo Soda, a superior preparation for nervous and certain other forms of headache, as we have repeatedly found to be the case in practice.

We invite attention to their advertisement in this Journal, which fully describes this medicine with a number of others prepared by this House—giving a number of useful formula, and interesting facts in regard to their Parvules, etc. We thank Messrs. Warner & Co. for the unique Christmas token in the shape of a beautifully ornamented knife, known as the Ingluvius knife.

DR. WILLIAM A. HAMMOND'S SANITARIUM for the Diseases of the nervous system was but recently established and had before its opening a very large number of applicants for admission, showing the great popularity of the Doctor, and that such an Institution as he has inaugurated was needed in Washington City, where it is located. See Doctor Hammond's advertisement in our Journal.

REED & CARNRICK.—This well known and reliable House are getting out some most excellent preparations. Be sure to examine their advertisement in this number of our Journal relating to *Liquid Peptonoids Pancrobilin* and *Phos-*

*pho-Caffein Comp.* The first has the remarkable property of dissolving diphtheretic deposit. So the other two preparations have very important proper. ties.

PERSONAL.—Mr. Herman Hasslock, of the firm of Hasslock & Ambrose, business manager of the Nashville Journal of Medicine and Surgery, is spoken of for the position of Public Printer under the administration of President Harrison. We have not the pleasure of a personal acquaintance with him, but from what we have heard of his promptness and business energy and his thorough knowledge in this particular line, we think his appointment would be eminently wise and judicious.

SYRUP OF TRIFOLIUM COMPOUND.—This is an alterative and blood remedy of superior properties. Especially suited to scrofulous and syphilitic affections. The ingredients which enter into its composition plainly indicate its adaptation to the class of cases mentioned. Each fluid ounce contains the active constituents of 32 grams of Red clover—16 grains of Stillingia, Burdock root, Poke root, Berberis Aquifolium and Cascara Amarga, 4 grams of Prickly Ash bark with 8 grains of Potassium Iodide. Prepared by Parke, Davis & Co., Detroit, Mich.

WRITING FOR THE JOURNAL.—We have promise of contributions from a number of good writers for the present year, and we again invite our readers generally to write something from their experience for the Record. We prefer short practical articles, as of reports of interesting cases, abstracts of society discussions, useful formulæ, etc. Almost every practitioner knows something not perhaps known by others. It may be a discovery—a practical fact developed in the emergencies of practice, or a new formula or combination of remedies which time and experience has shown to be efficient, and which if published would constitute an important addition to the armamentarium of the practitioner. Let every man who knows anything whatever which would be in any degree new or useful give it to the profession. By so doing you will accomplish some good for your fellow men and add your quota to the progress of Medical science in your section.

ALA. MED. & SURG. AGE.—We find on our table No 1. Vol. I, of a new publication, with the above title and edited by Dr. Jno. C. LeGrand of Anniston, Ala. A neatly published Journal of 26 pages oc. The first number makes a favorable impression as to the ability of the editor and the taste and excellence of the get up of the Journal. We place you on our exchange list brother LeGrand and wish you success in the laudable enterprise.

Cash recipts for 1888, Drs. R. H. Edwards; J. C. Moody to July; Allison Lockwood; E. Y. Flemming; W. S. Glass; J. I. Groover; J. A. Dobbins; A. R. Stephens; Robert A. Smith; Elias Swan; George Lathrop; William Simms; P. A. Ryan; R. S. Muller; J. A. Hyts; R. R. Jones.

For 1889, L M. Nichols; M. M. M. Evans to July; John Gerdine; J. C. Smith; W. S. Kendall; J. T. Lee; D. N. Nichols to June; T. H. Parham; S. M. Hogan; George Wyatt; C. Hamilton; Thos. Knott; Robt. Miller.

MEDICAL MISIONARY JOURNAL is a journal published in Chicago. It is the authorized Journal of the Medical Missionary Society. Edited by Rev. Wm. C. Stoudemise, A. M. Assisted by a number of Medical men of the City. It is a 32 page Monthly. Terms, $1 00 per annum. Devoted to the medical missionary work throughout the world. It is filled with articles of interest relating to the success attending the union of the practice of medicine with the work of missions, etc. It should be encouraged as a worthy and useful enterprise.

THE CASE OF EMPEROR FREDERICK III.—Full of official reports of the German Physicians, and by Sir Morrell McKenzie. The reports of the German Physicians translated by Henry Schweig, M. D., New York, 48 University Place. Edgar S. Warner, 1888. We have here a book of 275 pages in two parts. Part 1st contains the reports of the German Physicians. Part 2nd the report of Sir Morrell McKenzie.

No less than twelve German Physicians gave written reports in the first part of the work. Those who desire to see both sides of the controversy and acquaint themselves with all the facts connected with it can do so by reading this book.

